# Autoimmunity Related to Adipsic Hypernatremia and ROHHAD Syndrome

**DOI:** 10.3390/ijms23136899

**Published:** 2022-06-21

**Authors:** Akari Nakamura-Utsunomiya

**Affiliations:** 1 Department of Genetic Medicine, Hiroshima University Graduate School, 1-2-3 Kasumi, Minami-ku, Hiroshima 734-8511, Japan; akari-ped@hiroshima-u.ac.jp; 2 Department of Pediatrics, Hiroshima University Graduate School, 1-2-3 Kasumi, Minami-ku, Hiroshima 734-8511, Japan; 3 Division of Neonatal Screening, National Center for Child Health and Development, 2 Chome-10-1 Okura, Setagaya, Tokyo 157-8535, Japan

**Keywords:** autoimmunity, subfornical organ, ROHHAD, Na_x_, ZSCAN1, paraneoplastic syndrome

## Abstract

Specific antibody responses to subfornical organs, including Na_x_ antibody, have been reported in patients with adipsic hypernatremia of unknown etiology who do not have structural lesions in the hypothalamic–pituitary gland. The subfornical organ, also referred to as the window of the brain, is a sensing site that monitors sodium and osmotic pressure levels. On the other hand, ROHHAD syndrome is a rare disease for which the etiology of the hypothalamic disorder is unknown, and there have been some reports in recent years describing its association with autoimmune mechanisms. In addition, abnormal Na levels, including hypernatremia, are likely to occur in this syndrome. When comparing the clinical features of adipsic hypernatremia due to autoimmune mechanisms and ROHHAD syndrome, there are similar hypothalamic–pituitary dysfunction symptoms in addition to abnormal Na levels. Since clinical diagnoses of autoimmunological adipsic hypernatremia and ROHAD syndrome might overlap, we need to understand the essential etiology and carry out precise assessments to accurately diagnose patients and provide effective treatment. In this review, I review the literature on the autoimmune mechanism reported in recent years and describe the findings obtained so far and future directions.

## 1. Introduction

Adipsic hypernatremia was proposed in a commentary article introducing a case analysis paper by Avioli et al. [1]. In some publications, it may also be referred to as hypernatremia with thirst central disorder and reset of the osmostat [2,3]. This disorder has a heterogeneous clinical etiology, but its main characteristic signs and symptoms are hypernatremia, hypotonic urine accompanied by a decrease in antidiuretic hormone, and lack of thirst [3]. In some cases, the hypothalamus and osmotic sensors are damaged, including an innate structural disorder (e.g., septo-optic hypoplasia), a tumor in the hypothalamic–hypophysis region, and inflammation (e.g., encephalitis due to CMV, HHV6, or COVID-19), as reported recently [4,5,6,7,8,9,10]. An etiology involving the autoimmune process, specifically related to anti-subfornical organ antibody (anti-SFO) and anti-Na_x_ antibody, has also been reported [11,12].

ROHHAD (rapid-onset obesity with hypoventilation, hypothalamic, and autonomic dysregulation) syndrome was proposed by Ize-Ludlow et al. in 2007 [13]. A later age of onset (median age of 3 years), very dramatic sudden weight gain, and negative *PHOX2B* genotypic sequencing distinguish ROHHAD from congenital central hypoventilation syndrome (CCHS), which was first reported by Robert Mellins et al. in 1970 [14]. In a case with a neuroendocrine tumor (NET), it was described as ROHHAD-NET syndrome [15]. ROHHAD syndrome typically presents with rapid-onset obesity from about 3 years of age on average, hypophysis symptoms (including growth hormone and central hypothyroid and hypothalamic symptoms, including central apnea and Attention-Deficit Hyperactivity Disorder), and autonomic nervous system symptoms [16]. The clinical presentation of dysnatremia is associated with ROHHAD syndrome. It is a rare disease with a reported risk of sudden death. Some of its causes have been investigated, mainly including genetic factors (e.g., imprinting abnormalities) and autoimmune factors that have been suggested since around 2019 [17,18,19,20]. Recently, autoantibodies to ZSCAN1 have been detected in patients with ROHHAD-NET [21]. Rituximab therapy has been administered to ROHHAD patients [20]. In this paper, I aim to review the autoimmune pathophysiology common to both syndromes from the literature to date.

## 2. Overview of Adipsic Hypernatremia and ROHHAD Syndrome

### 2.1. Adipsic Hypernatremia

In healthy adults, plasma osmolarity and its principal determinant, plasma sodium, are usually maintained within a relatively narrow range, from 275 to 295 mOsm/kg and 135 to 145 mEq/L [22]. As consistency in serum sodium levels is essential for life support, interactions among thirst, drinking water intake, nerve arginine vasopressin (AVP) secretion, and the renal distal tubule are involved in the regulation [23,24].

Regarding adipsic hypernatremia, it has been proposed that the threshold level at which AVP secretion and thirst are stimulated is reset to a level higher than normal [23,24], and that the brain is involved in this pathological condition. Dysfunction in circumventricular organs (CVOs) with sodium sensor (Na_x_ channel) and osmotic pressure sensor (TPPV4 channel) functions leads to resetting the osmostat level to an abnormally high range [25]. There are three parts of CVOs, namely, the subfornical organ (SFO), the organum vasculosum of the lamina terminalis (OVLT), and the area postrema [26]. Among them, the SFO plays a major role as a sodium sensor [26]. The SFO is the center of brain thirst and salt-craving control and is also involved in antidiuretic hormone (ADH) secretion control [27]. In addition, this site is a blood vessel-rich site that escapes the blood–brain barrier and is in direct contact with cerebrospinal fluid [27]. It is also known that antibodies invade CVOs because they do not have blood–brain barrier restrictions [26].

Adipsic hypernatremia is thought to be caused by such factors as a congenital lack of CVOs, tumors, abnormal blood flow, and damage due to immune responses. The factors reported to be the cause of this disease are shown in Table 1. In addition, Na_x_ is expressed in the SFO site and is reported as a sodium sensor; one case with ganglioneuroma was reported, which was a case of a girl who was positive for anti-Na_x_ antibody [11], although the cause was unknown. There is also a report that a specific antibody reaction to mouse SFO is observed in this disease [12].

Among CVOs, the SFO is a site that plays a major role as a sodium sensor and an osmoreceptor and is considered to be the center for thirst and salt desire control in the brain [25,26]. In addition, the SFO has afferent and efferent neurons with the surrounding hypothalamic nucleus and has a mutual network. Therefore, it is assumed that various hypothalamic and pituitary dysfunctions will occur if the same site is absent or damaged [26]. Although the clinical picture of this disease is widely observed, the main process of decreased AVP secretion is a neuroprojection because the SFO has efferent neurons that project to the paraventricular nucleus and is involved in the regulation of ADH secretion. The decrease is accompanied by a decrease in ADH secretion. Essential hypernatremia is likely to be accompanied by a partial decrease in ADH.

### 2.2. ROHHAD Syndrome

ROHHAD syndrome is defined and diagnosed from clinical symptoms such as hypothalamic symptoms with rapid-onset obesity in early childhood, pituitary hormonal disorders, and autonomic symptom disorders [31,32]. The symptoms resulting from the lesion suggest that it is associated with hypothalamic disorders. The previous literature has also described it as Ondine’s curse because it is a progressive, lethal condition of unknown etiology [33]. All cases exhibited sudden obesity and central hypoventilation. Frequent complications are growth hormone deficiency and hypernatremia, and the complications seen in half of the patients are hyperprolactinemia, central hypothyroidism, and cardiac/respiratory arrest. Differentiation from CCHS is required for this syndrome, but gastrointestinal or ocular symptoms may not be observed in this disease. As for what causes the hypothalamic disorder, genetic factors and autoimmune factors have been reported so far [15,16]. Genes involved in the development of the hypothalamic–pituitary gland and related to the sleep cycle are listed. However, it is still uncertain as to whether any of these genes are disease-related. The *PHOX2B* gene is a transcriptional factor and related to the regulation of neural crest movement and the development of the autonomic nerve system. The *PHOX2B* variant is the point for differentiation between ROHHAD syndrome and CCHS. In a paper comparing the clinical findings of ROHHAD syndrome and Prader–Willi syndrome, which is similar to hypothalamic obesity, three points are listed as distinguishing points [29]. First, the presence or absence of symptoms at birth, the presence or absence of abnormal water metabolism, including Na levels, and the presence or absence of hyperprolactinemia are mentioned [29]. On the other hand, some autoimmunity and acquired factors, such as anti-SFO, anti-hypothalamus, and anti-ZSCAN1 autoantibodies, can be inferred in addition to the genetic predisposition [21,28,30].

## 3. Findings on the Similar Pathogenic Mechanism in the Two Disease Groups 

Recent reports show that autoantibodies have been detected in both diseases, and autoimmunity is speculated as a new mechanism of pathogenesis. Adipsic hypernatremia is thought to be caused by such factors as a congenital lack of CVOs, tumors, abnormal blood flow, and damage due to immune responses [4,5,6,7,8,9,10,11,12]. The factors reported to be the cause of this disease are shown in Table 1. In addition, Na_x_ is expressed in the SFO site and is reported as a sodium sensor; one case of a girl with anti-Na_x_ antibody positivity and a ganglioneuroma was reported [11]. There is a report that a specific antibody reaction to mouse SFO is observed in this disease [12]. This pathophysiology might have been due to complement-mediated cell death in the Na_x_-positive regions because deposits of injected IgG proteins and the C3 component of the complement system were specifically observed in the SFO and OVLT [11]. The classical complement pathway proceeds to eventually produce the membrane attack complex (MAC) to lyse the cellular membrane [34]. The death of these glial cells may have caused the dysfunction of neurons in the SFO and OVLT. Furthermore, apoptosis in the SFO and OVLT resulted in the lack of thirst sensation, regardless of hypernatremia.

**Figure 1:**
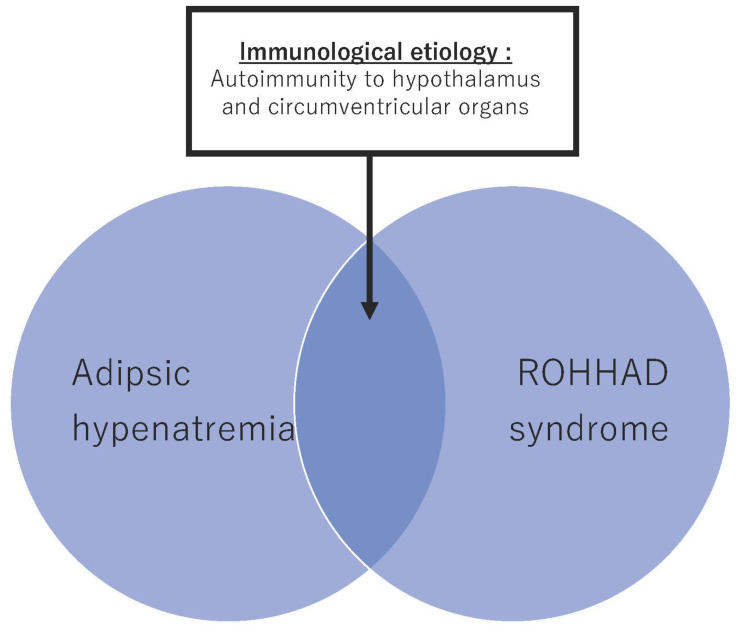
Concept of autoimmunological adipsic hypernatremia and ROHHAD syndrome.

As it is suggested that periventricular organs and the hypothalamus each have a network, it is assumed that injuries to each of these may cause similar medical conditions. Interestingly, recent reports have identified antibodies to SFO and Na_x_ antibodies in patients with clinically diagnosed autoimmune hypernatremia and ROHHAD syndrome. From this, it is possible that some patients in both disease groups develop the disease by a common autoimmune mechanism (Figure 1) [28].

There have been some recent reports supporting the autoimmune mechanism in ROHHAD syndrome. Cases associated with celiac disease, an association between tumor immunity and ganglioneuroma, and a recent report about the anatomical findings of ROHHAD syndrome showed encephalitis (lymphocyte infiltration) findings, including around the ventricles [18]. According to the autopsy report, hypothalamic infiltrates involved multiple regions, including the anterior, paraventricular, arcuate, and ventromedial nuclei [18]. There are also reports of the identification of anti-Na_x_ and anti-SFO antibodies in patients diagnosed with ROHHAD syndrome [28]. It has also been reported that the prolactin level was significantly higher in antibody-positive patients, regardless of the clinical diagnosis, and it is considered that the hyperprolactinemia observed in ROHHAD syndrome so far is also consistent with this concept [28].

## 4. Relationship between the Hypothalamus and Osmoreceptors in Periventricular Organs

The hypothalamus can be considered the coordinating center of the endocrine system. It consolidates signals derived from upper cortical inputs, autonomic function, environmental cues such as light and temperature, and peripheral endocrine feedback [35]. In turn, the hypothalamus delivers precise signals to the pituitary gland, which then releases hormones that affect most endocrine systems in the body. From an anatomical point of view, the hypothalamus is located at the base of the brain, below the third ventricle and just above the optic chiasm and pituitary gland [36,37]. This location can be thought of as the intersection of the cortex, the cerebellum, and the brainstem. Most of the cell bodies of small neurons containing hypothalamic-releasing hormones are located in the tuberal area in the anterior part of the hypothalamus [37]. Afferent pathways to hypothalamic nuclei arise from the brainstem, thalamus, basal ganglia, cerebral cortex, and olfactory areas. Efferent pathways include the dorsal longitudinal fasciculus connecting the hypothalamus to the brainstem reticular centers, connections to the autonomic nervous system and the thalamus, and the hypothalamo-neurohypophysial tract that connects the paraventricular and supraoptic nuclei, which produce ADH, to nerve terminals in the median eminence and to the posterior pituitary [38,39].

CVOs include only the median eminence and adjacent neurohypophysis, OVLT, SFO, and the area postrema. The SFO and OVLT are the parts of the circumventricular organ that lack the blood–brain barrier restriction and allow vascular molecules to permeate the brain parenchyma. It is also called the “brain window”. The SFO extends efferent axonal projections to the median preoptic nucleus (MnPO), OVLT, supraoptic nucleus, arcuate nucleus, lateral preoptic area, and lateral hypothalamus. A small portion of SFO neurons in the periphery extend collateral projections to both the MnPO and the paraventricular nucleus of the hypothalamus, likely affecting the AVP system [26].

It is associated not only with the AVP metabolic system but also with efferent nerves from the SFO and the appetite center. Orexigens and anorexigens both act at the SFO, but via different neuronal pathways [40]. Some experimental evidence suggests that ghrelin may play a role in the regulation of energy balance by action at the SFO [41]. Accordingly, information sensed by the SFO as sensors, such as sodium in the cerebrospinal fluid, osmotic pressure, and LPS, affects the nerve nucleus existing in the hypothalamus through each nerve fiber [41]. Therefore, a mechanism by which the hypothalamic disorder develops due to damage might be assumed.

## 5. Clinical Symptoms and Complications

In autoimmune hypernatremia, there is polyuria, including nocturnal urination, but the feeling of dry mouth is lacking or diminished, and proper drinking behavior is not observed [26]. Hyperhidrosis, cold limbs, and mild dehydration may occur [1,2]. In addition to an increase in serum Na levels and serum osmolality, an increase in prolactin levels, an increase in the frequency of hypothalamic disorders, and an increase in retroperitoneal tumors have been reported [28]. Moreover, our study revealed that plasma renin activity was significantly higher in the anti-Nax antibody-positive cases. In cases with hypothalamic syndrome, rapid-onset obesity, weight gain tendency with or without overeating, and insomnia symptoms are observed; some cases are also diagnosed with Attention-Deficit Hyperactivity Disorder [28]. There is also a complication of sleep apnea syndrome due to obesity. If sodium levels are high, convulsions, impaired consciousness, weakness, and other signs and symptoms are exhibited. These hypothalamic symptoms are also symptoms encountered in ROHHAD syndrome.

Symptoms of hypopituitarism are observed when accompanied by either disorder. In the cases reported in the literature, central hypothyroidism, central adrenal gland dysfunction, decreased growth hormone secretion, central precocious puberty, central hypogonadism, and so forth are observed, and these may be complicated. Characteristic laboratory findings common to both disease groups include abnormal sodium levels and hyperprolactinemia [28]. It is known that the arcuate nucleus, which has a neural network, secretes dopamine, which is a prolactin inhibitor, from the SFO. Damage caused by antibodies to SFO indirectly lowers dopamine, resulting in high prolactin levels [31].

## 6. New Therapeutic Approach

The treatment is supplementation with water or AVP for fluctuations in Na levels and supplementation with various preparations for impaired pituitary hormones. On the other hand, for hypothalamic disorders in ROHHAD syndrome, only symptomatic treatment is available. For example, for obesity, a low-calorie diet and exercise encouragement are recommended [31,32]. For sleep apnea and central apnea, SpO_2_ monitoring, oxygen administration, and positive pressure ventilation at home have also been attempted [31,32].

Some immunosuppressive treatments have been reported for ROHHAD syndrome, for which an autoimmune mechanism has been reported. Lisa et al. reported that a case with ROHHAD showed improved behavior and neuropsychological function with high-dose cyclophosphamide (50 mg/kg/day) [19]. It was reported that it was effective in improving behavior and weight loss, and the effect was recognized even one year later. Recently, Hawton et al. reported a case treated with rituximab therapy [20]. According to the report, a girl had central hypoventilation, central diabetes insipidus, growth hormone deficiency, and hyperprolactinemia [20]. Elevated interleukin-6 levels were detected by cytokine serology, and the level normalized after rituximab treatment [20]. Furthermore, after rituximab treatment, her weight decreased significantly in 12 months. Accordingly, immunosuppressive therapy might be effective for improved outcomes. In the future, if the effects of early immunosuppressive treatment become clear through clinical trials, improved outcomes are expected, but there is no reliable disease monitoring, so it is considered to be a future task.

## 7. New Insight on Topics Related to Autoimmune Adipsic Hypernatremia and ROHHAD Syndrome

Three interesting and important reports have been made since the beginning of this year. One is the identification of a novel antigen called ZSCAN1 in ROHHAD cases with tumors, cases in which ROHHAD-like symptoms were observed after COVID-19 infection, and cases including adults with anti-Na_x_ and anti-SFO antibody responses in adipsic hypernatremia and ROHHAD syndrome. Mandel-Brehm et al. reported that ZSCAN1 autoantibodies were detected in patients with tumor-associated ROHHAD syndrome [21]. ZSCAN1 is a transcriptional factor and expressed in the hypothalamus [42]. On the other hand, regarding ZSCAN1, the kind of gene expression that it is related to as a transcription factor has not yet been clarified as a function. In the future, detailed pathophysiology focusing on antigens should become clear, and at the same time, its usefulness as an early diagnostic marker for diseases has been suggested.

The first description of ROHHAD-like syndrome was temporally associated with a previous COVID-19 infection with possible primary viral or immune-mediated hypothalamic involvement [10]. Furthermore, treatment with non-steroidal anti-inflammatory drugs and monthly courses of intravenous immunoglobulin led to a dramatic improvement. In addition to rituximab, IVIG therapy might be useful in avoiding disease progression [10].

Even in adult patients with autoimmune hypernatremia, cases were reported in which autoimmune mechanisms such as anti-SFO antibody reaction were assumed, and cases clinically diagnosed as ROHHAD were identified. If the disease can be confirmed by early diagnosis using biomarkers, such as antibody reactions as well as clinical features, early treatment is expected to be effective.

## 8. Conclusions and Future Directions

In recent years, autoimmune mechanisms have been reported for adipsic hypernatremia and ROHHAD syndrome, the causes of which were once unknown, and new findings allowing for early diagnosis and treatment are expected in the future. Patients have unmet needs, and it is hoped that the prognosis will be improved by appropriate management in the future. I hope that this paper will serve as a reference for the pathophysiology and disease process.

## Figures and Tables

**Table 1:** Etiology in adipsic hypernatremia and ROHHAD (NET) syndrome.

Cause	Adipsic Hypernatremia (Average Serum Sodium Level: 157.1 ± 15.2 mM in Pediatric and Adult Patients) (Ref. [28])	ROHHAD (NET) Syndrome (Dysnatremia: 45.5% (15/33 Cases), Shown in 5~10 Years Old) (Ref. [15])
Genetic or innate factor	Structural defect in central septal lesion of brain (septo-optic dysplasia, agenesis of cortex, etc. (Refs. [4,5]))	Some candidate genes (*PHOX2B*, *BDNF*, *RAI-1*, etc.) have been analyzed but not confirmed. (Ref. [15])
Associated Tumor	Craniopharyngioma, germinoma, etc., in hypothalamus and pituitary lesion	Neural crest tumor (neuroblastoma, ganglioblastoma, etc.)
Inflammatory factors	Viral encephalitis (CMV, HHV-6, or COVID-19) (Refs. [8,9,10])	Similar symptoms to COVID-19 (Ref. [10])
Immunological factors	Autoantibodies:	Autoantibodies:
	Anti-Na_x_ antibody Anti-subfornical antibody (Refs. [11,12,28])	Anti-hypothalamus, anti-pituitary Anti-subfornical antibody Anti-ZSCAN1(in ROHHAD-NET) (Refs. [21,28,29,30])
	Area:	Area:
	CVOs (SFO and OVLT) and their neural network area (SON, ARC, PVN, etc.) (Refs. [11,12,28])	Hypothalamus, brainstem, pontine tegmentum, midbrain, pons, upper cervical cord, thalamus (Ref. [18])
	Infiltrated or related immunological cells:	Infiltrated or related immunological cells:
	Deposition of the C3 component and infiltration of inflammatory cells Apoptosis in SFO and OVLT	Dense perivascular lymphocytic infiltrate CD20^+^ B-cells, patchy nodular parenchymal lymphocytic infiltrate CD8^+^ T-cells (Ref. [18])
